# A Role for Both V1a and V2 Receptors in Renal Heat Stress Injury Amplified by Rehydration with Fructose

**DOI:** 10.3390/ijms20225764

**Published:** 2019-11-16

**Authors:** Fernando E. García-Arroyo, Itzel Muñoz-Jiménez, Guillermo Gonzaga, Edilia Tapia, Horacio Osorio-Alonso, Carlos A Roncal-Jiménez, Alison Iroz, Mariacristina Vecchio, Juan G. Reyes-García, Richard J Johnson, L Gabriela Sánchez-Lozada

**Affiliations:** 1 Department of Cardio-Renal Physiopathology, INC Ignacio Chávez, Mexico City 14080, Mexico; jonibertojr@hotmail.com (F.E.G.-A.); itzel.morrison@icloud.com (I.M.-J.); ggonzaga49@gmail.com (G.G.); ediliatapia@hotmail.com (E.T.); horace_33@yahoo.com.mx (H.O.-A.); 2 Sección de Estudios de Posgrado e Investigación, Escuela Superior de Medicina, IPN Mexico City 11340, Mexico; juangreyesgarcia@gmail.com; 3 Renal Diseases and Hypertension, University of Colorado Anschutz Medical Campus, Aurora, CO 80045, USA; CARLOS.RONCAL@cuanschutz.edu (C.A.R.-J.); RICHARD.JOHNSON@cuanschutz.edu (R.J.J.); 4 Danone Research, 91767 Palaiseau, France; Alison.PETIT-JEAN@danone.com (A.I.); Cristina.VECCHIO@danone.com (M.V.)

**Keywords:** vasopressin, aldose reductase, fructokinase, angiotensin II, SGK1, cortisol

## Abstract

Chronic vasopressin secretion induced by recurrent mild heat stress exposure is significantly enhanced by limited rehydration with a fructose-containing beverage both in rodents and in humans. Moreover, this effect has been associated with upregulation of the polyol–fructokinase pathway and increased renal oxidative stress. Previously, we have shown that pharmacological inhibition of both V1a and V2 vasopressin receptors with conivaptan improved such renal alterations. The aim of this study was to evaluate the independent contributions of V1a and V2 receptors to the renal damage caused by mild heat stress and limited rehydration with a fructose-containing beverage. Osmotic minipumps were used to deliver either relcovaptan (0.64 mg/day) or tolvaptan (0.25 mg/day) in male Wistar rats for two weeks. Corresponding dilution vehicles were used as controls. To induce dehydration, rats were exposed to mild heat stress (37 °C for 1 h, Monday to Friday). All groups received a 10% fructose solution as a rehydration fluid for 2 h after mild heat stress. For the remainder of the day and on weekends, rats received tap water. The independent blockade of either the V1a or the V2 receptor prevented renal damage, reduced oxidative stress, and decreased plasma cortisol and systemic inflammation. However, the beneficial effects were regulated by different mechanisms. Tolvaptan inhibited polyol–fructokinase pathway overactivation, while relcovaptan prevented upregulation of the renin–angiotensin system and SGK1 expression. These data suggest that both V1a and V2 receptors participate in renal damage caused by heat stress-induced dehydration when fructose-containing beverages are used as rehydration fluids.

## 1. Introduction

The causes that originate and sustain the progression of chronic kidney disease (CKD) are incompletely understood. It is well agreed that diabetes and hypertension, which target the kidneys, are main causes of CKD worldwide [1]. However, it is currently recognized that environmental and lifestyle factors also contribute to kidney damage [2,3]. For example, heat stress, caused by heatwaves or extreme labor conditions, is one of the most catastrophic factors that threatens kidney health, especially in specific populations such as the elderly, renal patients, and children [4]. Dehydration induced by such conditions may cause recurrent episodes of prerenal acute kidney injury that may eventually evolve to CKD. Among the mechanisms of damage, chronic vasopressin secretion and overactivation of the polyol–fructokinase pathway have been recently investigated [5,6,7].

The possible role of chronic vasopressin secretion in the incidence and progression of diabetic and non-diabetic CKD has been recently highlighted in various clinical [8,9,10,11,12] and experimental studies [7,13]. Chronic stimulation and/or overexpression of vasopressin receptors V1a and V2 have been associated with renal and cardiac alterations, while chronic blockade of both receptors has provided beneficial effects [14,15,16]. Moreover, there is evidence that increased serum osmolality, a strong stimulator of vasopressin secretion, is an independent risk factor for developing CKD [17]. In addition, there are non-osmotic stimulators that might perpetuate a state of chronic vasopressin secretion. For example, fructose induces vasopressin secretion by a mechanism that includes its metabolism by fructokinase in hypothalamic explants [18,19]. Thus, chronic vasopressin secretion induced by recurrent mild heat stress exposure was significantly enhanced by limited rehydration with a fructose-containing beverage, both in rodents and in humans [6,7,20], further aggravating kidney damage. Increased plasma osmolality may also augment fructose production by stimulating the nuclear translocation of NFAT5, which is the main regulator of aldose reductase expression, the limiting enzyme of the polyol pathway [21]. In turn, fructose metabolism by fructokinase overexpression results in a positive feedback upregulation of both fructokinase and aldose reductase, thus amplifying this pathway [5,22]. 

Another potential mechanism of renal damage induced by dehydration is the overexpression of serum- and glucocorticoid-inducible kinase 1 (SGK1), which is upregulated by both vasopressin and NFAT5 nuclear translocation. Increased activity of SGK1 has been associated with the physiopathology of hypertension and cardiac and renal failure [23]. 

We previously reported that blocking both V1a and V2 vasopressin receptors with conivaptan prevented the overexpression of aldose reductase, sorbitol dehydrogenase, and fructokinase in mildly dehydrated rats rehydrated with a fructose-containing beverage [7]. Such effects were associated with the prevention of renal functional changes and oxidative stress.

Vaptans are non-peptide vasopressin receptor antagonists. Relcovaptan (SR49059, V1a blocker), tolvaptan (OPC-41061, V2 blocker), and conivaptan (YM-087, V1a/V2 blocker) belong to this family of drugs. 

Therefore, the aim of this study was to evaluate, in mildly heat-stressed rats rehydrated with limited fructose-containing beverage, the contribution of V1a and V2 receptors to renal damage by blocking each receptor independently with relcovaptan and tolvaptan, respectively.

## 2. Results

The mean intake of 10% fructose beverage after heat stress or during the two hours it was available to non-heat-exposed rats, was not different among the groups. In addition, the total fluid intake over 24 h (including the 10% fructose fluid and tap water the rest of the day) was not different among the groups (Table 1). Body weight loss after heat-induced dehydration was 2–3% in all heat-exposed groups, and the tolvaptan groups tended to have higher weight loss values (*p* < 0.05 vs. relcovaptan); however, a multiple comparison test resulted in non-significant differences among the groups (data not shown). One rat, in group 5, H-TV, died for reasons not related to the experimental protocol.

### 2.1. Relcovaptan and Tolvaptan Conferred Nephroprotection in HeatStress-Dehydrated Rats Rehydrated with a 10% Fructose Beverage

**Figure 1:**
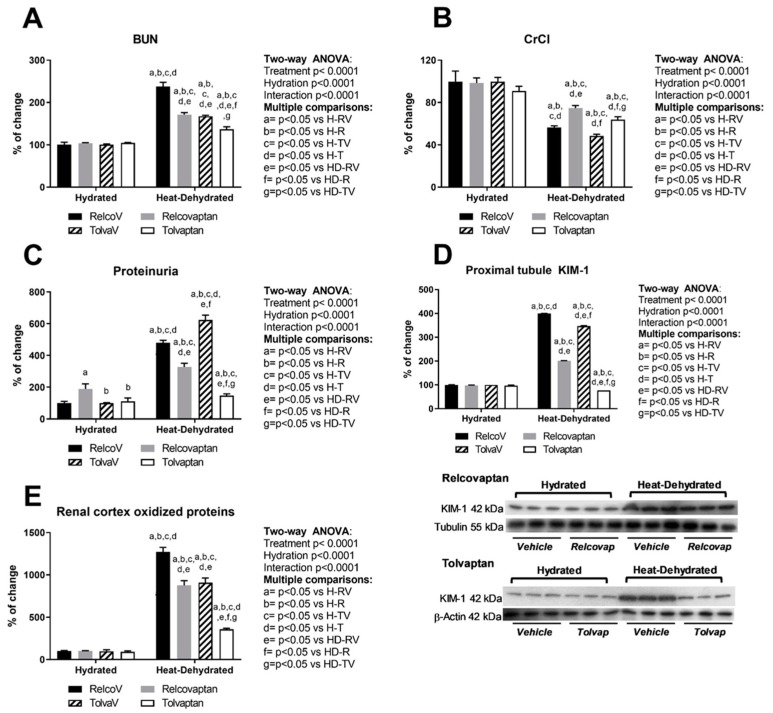
Relcovaptan and tolvaptan conferred nephroprotection in heat stress-dehydrated rats rehydrated with a 10% fructose beverage. (**A**) Blood urea nitrogen (BUN) was increased by heat stress and rehydration with a fructose beverage; relcovaptan and tolvaptan treatments prevented this effect. (**B**) Creatinine clearance (CrCl) was significantly decreased by heat stress and rehydration with a fructose-containing beverage; relcovaptan and tolvaptan treatments partially prevented this effect. (**C**) Proteinuria, (**D**) proximal tubule KIM-1 expression, and (**E**) oxidative stress were increased by heat stress and fructose beverage rehydration. Relcovaptan and tolvaptan treatments prevented such effects, with tolvaptan being more effective. For western blotting, three random samples per group were selected. Proteins of interest and the respective loading controls were run independently at the same time using the same conditions. The raw dataset is available in the Supplementary Materials.

Neither relcovaptan nor tolvaptan had any effects on the kidney parameters examined in non-heat-exposed animals rehydrated with fructose, the only exception being urine protein excretion that was mildly increased by relcovaptan but still within normal range (Figure 1). On the other hand, heat-induced dehydration with fructose rehydration increased blood urea nitrogen (BUN, Figure 1A), decreased creatinine clearance (CrCl) (Figure 1B), and elevated proteinuria (Figure 1C), KIM-1 expression (Figure 1D), and renal oxidative stress (Figure 1E). Importantly, both relcovaptan and tolvaptan treatments had beneficial effects, with tolvaptan being more effective at reducing proteinuria, KIM-1 expression in the proximal tubule, and oxidative stress. Two-way ANOVA analysis showed statistical significance by treatment, hydration state, and the interaction between the two factors for all these parameters (Figure 1).

### 2.2. Plasma Copeptin and Vasopressin Receptors Expression in the Renal Cortex 

**Figure 2:**
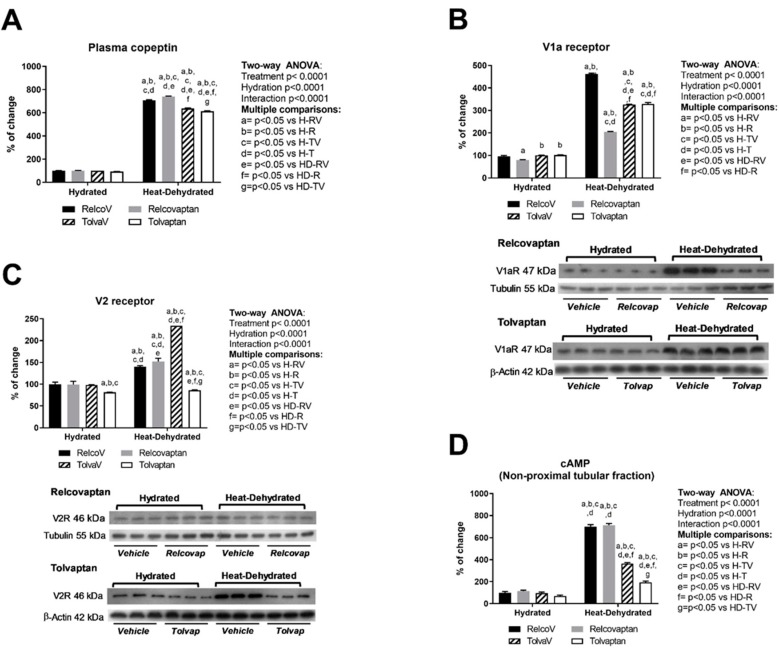
Plasma copeptin and vasopressin receptors expression in the renal cortex. (**A**) Plasma copeptin was increased in heat-stressed rats rehydrated with the fructose beverage, regardless of relcovaptan or tolvaptan treatment. V1a receptor (**B**) and V2 receptor (**C**) expressions were increased by heat stress and fructose beverage rehydration. Relcovaptan partially prevented V1a overexpression (**B**), and tolvaptan fully prevented V2 overexpression (**C**). (**D**) Likewise, only tolvaptan prevented the increment of cAMP in the non-proximal tubule fraction induced by heat stress and rehydration with the fructose beverage. For western-blotting, three random samples per group were selected. Proteins of interest and the respective loading controls were run independently at the same time using the same conditions. The raw dataset is available in the Supplementary Materials.

All heat-dehydrated groups rehydrated with a fructose-containing beverage had a six- to sevenfold elevation in vasopressin secretion (as measured by plasma copeptin) compared with the respective non-dehydrated groups (Figure 2A). Two-way ANOVA showed statistical significance by treatment, hydration state, and the interaction between the two factors (Figure 2A). Figure 2C,D shows that treatment with tolvaptan blocked the expression of the V2 receptor and decreased its downstream signaling molecule, cAMP. No effect on V1a receptor expression was observed in the tolvaptan groups (Figure 2B). Relcovaptan only blocked V1a receptor expression, showing that the agents were specific for their targets (Figure 2B).

### 2.3. Effect of Heat Stress and Rehydration with a Fructose-Containing Beverage on Systemic Inflammation and Plasma Cortisol Levels

Heat-induced dehydration with fructose rehydration elevated plasma IL-6 in all groups. Both relcovaptan and tolvaptan partially prevented this effect, but tolvaptan was significantly more effective (relcovaptan—20%, tolvaptan—33%). Two-way ANOVA analysis showed statistical significance by treatment, hydration state, and the interaction between the two factors (Table 2).

Plasma cortisol was also increased (9- to 15-fold) by heat stress and rehydration with the 10% fructose beverage (Table 2). Independent blockade of either V1a or V2 receptors provided benefit, but only blocking of the V1a receptor by relcovaptan fully prevented this effect.

### 2.4. Tolvaptan Prevented the Overexpression of Polyol Pathway Enzymes (Aldose Reductase and Sorbitol Dehydrogenase) and Fructokinase in Heat Stress-Dehydrated Rats Rehydrated with a 10% Fructose Beverage

**Figure 3:**
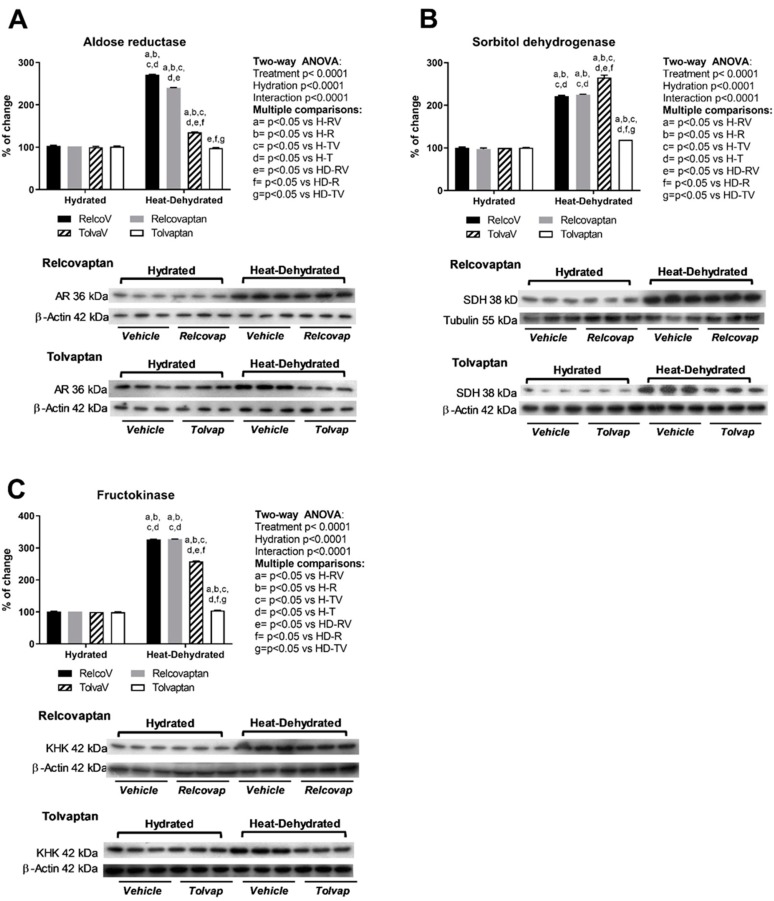
Effects of tolvaptan on polyol and fructokinase pathways. Tolvaptan prevented the overexpression of (**A**) aldose reductase, (**B**) sorbitol dehydrogenase, and (**C**) fructokinase in heat stress-dehydrated rats rehydrated with a 10% fructose beverage. For western blotting, three random samples per group were selected. Proteins of interest and the respective loading controls were run independently at the same time using the same conditions. The raw dataset is available in the Supplementary Materials.

Aldose reductase (Figure 3A), sorbitol dehydrogenase (Figure 3B), and fructokinase (Figure 3C) expressions were increased by heat-induced dehydration and rehydration with fructose. Tolvaptan treatment prevented the overexpression of sorbitol dehydrogenase and fructokinase. Relcovaptan had a mild effect on aldose reductase overexpression (−12%), while tolvaptan fully prevented this effect. Two-way ANOVA analysis showed statistical significance by treatment, hydration state, and the interaction between the two factors for all these parameters (Figure 3).

### 2.5. Relcovaptan Protected Against the Overactivation of the Renin–Angiotensin System in Heat Stress-Dehydrated Rats Rehydrated with a 10% Fructose Beverage

**Figure 4:**
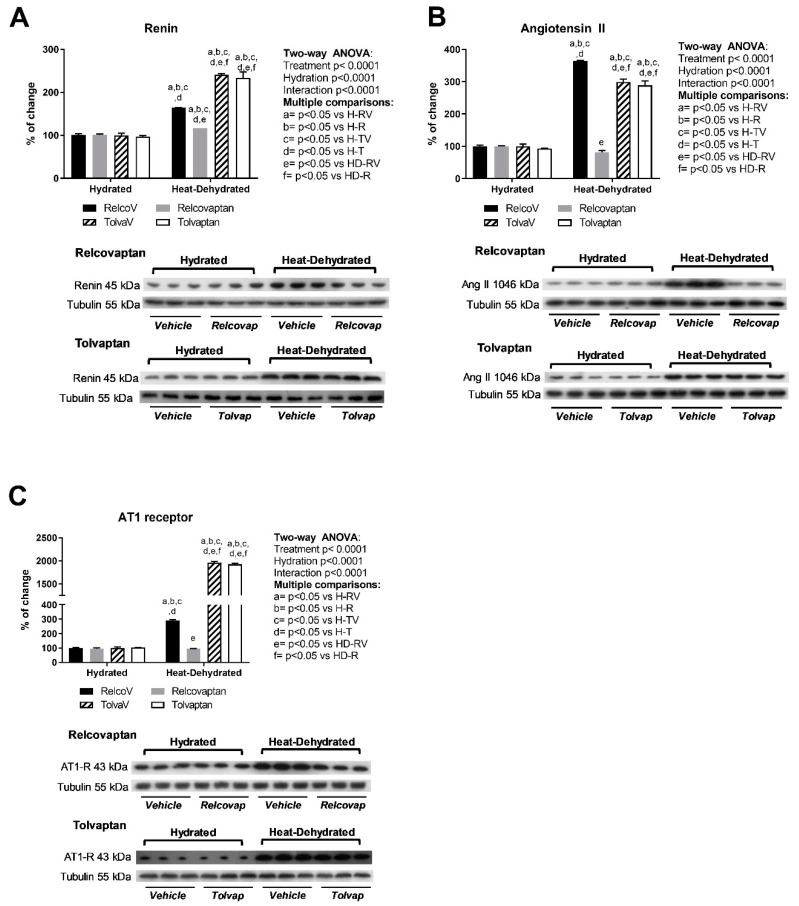
Effects of relcovaptan on the renin–angiotensin system. Heat stress and rehydration with a fructose-containing beverage induced the overexpression of renin (**A**), angiotensin II (**B**), and AT1 receptor (**C**). Tolvaptan prevented such effects. For western blotting, three random samples per group were selected. Proteins of interest and the respective loading controls were run independently at the same time using the same conditions. The raw dataset is available in the Supplementary Materials.

Heat stress and rehydration with the fructose beverage induced the overexpression of renin (Figure 4A), angiotensin II (Figure 4B), and AT1 receptor (Figure 4C). Treatment with relcovaptan prevented such effects. In contrast, tolvaptan had no effect. Two-way ANOVA analysis showed statistical significance by treatment, hydration state, and the interaction between the two factors for these parameters.

### 2.6. Relcovaptan Prevented the Overexpression of Glucocorticoid-Inducible Kinase 1 (SGK1) in Heat Stress-Dehydrated Rats Rehydrated with a 10% Fructose Beverage

**Figure 5:**
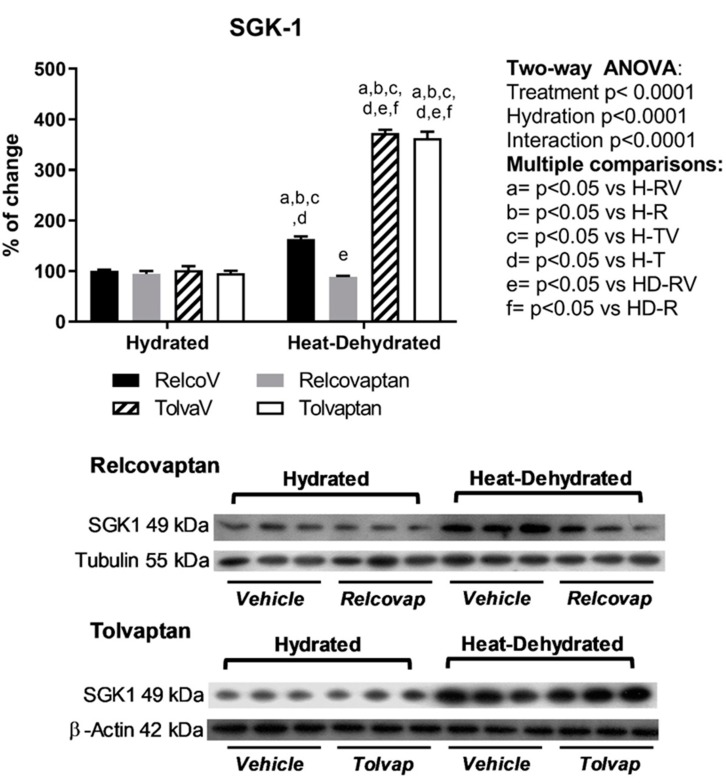
Effects of relcovaptan and tolvaptan on glucocorticoid-inducible kinase 1 (SGK1). Relcovaptan prevented the overexpression of SGK1 in heat stress-dehydrated rats rehydrated with a 10% fructose beverage. For western blotting, three random samples per group were selected. Proteins of interest and the respective loading controls were run independently at the same time using the same conditions. The raw dataset is available in the Supplementary Materials.

Heat stress and rehydration with a fructose-containing beverage increased the renal cortex expression of SGK1 (1.6–3.7-fold increase) (Figure 5). Only relcovaptan prevented this effect. Two-way ANOVA analysis showed statistical significance by treatment, hydration state, and the interaction between the two factors for these two parameters.

## 3. Discussion

The present study showed that both V1a and V2 receptors participate in the renal alterations induced by heat stress and rehydration with fructose-containing beverages. The independent blockade of each receptor prevented renal damage, reduced oxidative stress, and decreased plasma cortisol and systemic inflammation, although a more potent effect was observed with tolvaptan.

Some divergent effects were induced by the blockade of V1a and V2 vasopressin receptors. Thus, the additional benefit provided by tolvaptan may be related to its effect of preventing the overexpression of the polyol–fructokinase pathway, whereas this effect was not observed with relcovaptan. Unraveling the mechanisms by which V2 receptor activation induces polyol–fructokinase enzymes overactivation is beyond the scope of this study. However, there is evidence that protein kinase A (PKA) activation plays an important role in the transactivation of the transcription factor tonicity-responsive enhancer/osmotic response element-binding protein (TonEBP/NFAT5), as H89 (a PKA inhibitor) significantly reduced the hypertonic induction of aldose reductase [24]. Therefore, these findings suggest that the lack of PKA activation mediated by V2 receptor antagonism with tolvaptan might prevent aldose reductase overexpression induced by heat stress dehydration and rehydration with a fructose-containing beverage. On the other hand, the prevention of fructokinase overexpression mediated by tolvaptan might have resulted from a greater reduction of oxidative stress, as it has been shown that antioxidants prevent fructokinase overexpression in heat-stressed rats rehydrated with a fructose-containing beverage [25].

It has been demonstrated that the renin–angiotensin system (RAS) can be regulated through the activation of the V1a receptor. Specifically, renin expression was suppressed in V1a receptor knock-out mice [26]. A reduction in vascular volume induced by heat-induced dehydration likely also activates RAS, and indeed in the present study, we observed an increased expression of RAS components in the renal tissue of heat-dehydrated rats. V1a receptor blockade accomplished by relcovaptan partially prevented the overexpression of renin, angiotensin II, and the AT1 receptor in renal cortex tissue. This effect likely provided therapeutic benefit in these rats. In addition, the V1a receptor blockade also prevented SGK1 renal overexpression, which was likely secondary to the prevention of angiotensin II overactivation [27].

Vasopressin increases cortisol secretion through adrenocorticotropic hormone (ACTH) stimulation mediated by the activation of V1b receptors at the pituitary level [28]. In the present study, both tolvaptan and relcovaptan decreased cortisol plasma concentrations. This finding suggests that both blockers might also have inhibitory effects on the V1b receptor or that cortisol secretion was influenced by other pathways affected by these antagonists. However, a definitive explanation for this effect needs to be further investigated.

Some limitations of our study should be stated. One should be cautious when extrapolating animal studies to human situations. This study had a relatively short-term follow-up; therefore, we do not know whether the conclusions could apply to more extended-duration studies. There were also differences in the expression of the examined markers between the relcovaptan and the tolvaptan groups; this could be attributed to the fact that the samples from each drug treatment were run independently, and such variations could be due to the use of different blots for expression evaluation. We decided to present the data from the two treatments together to accomplish an impartial comparison between them. Despite this, the outcomes resulting from relcovaptan and tolvaptan treatments in heat-dehydrated groups remain valid. 

Overall, the results of the present study are in agreement with those of other models of renal damage, in which chronic blockade of V1a and V2 receptors have provided renoprotection [14,15,16]. Despite relcovaptan and tolvaptan not being fully specific V1a and V2 receptors antagonists [29,30], these data suggest that both receptors are implicated in the renal damage caused by heat stress-induced dehydration when fructose-containing beverages are used as rehydration fluids. Moreover, the combined blockade of V1a and V2 receptors may be superior to their individual blockade, as we previously demonstrated using conivaptan therapy [7]. These results may have clinical relevance, as there is evidence that the intake of plain water is below the recommendations stated by the Institute of Medicine and the European Food Safety Authority’s current guidelines [31], especially in children, in whom there is a high prevalence of inadequate total water intake [32,33,34,35]. In addition, the strong preference for the sweet taste [36], together with aggressive advertisement campaigns for sweetened beverages [37], have greatly increased the intake of sugary beverages with high fructose content [38]. Therefore, such behaviors may be detrimental for kidney health. These data also shed light on the nephroprotective potential of blocking V1a and V2 vasopressin receptors in specific populations that are continuously exposed to heat stress and strenuous work, such as labor and industrial workers, soldiers, miners, firefighters, etc.

## 4. Materials and Methods

These studies were conducted with strict adhesion to the current international guidelines for laboratory animal work and care and were approved by the Internal Animal Care and Use Committee (Permit No INC/CICUAL/005/2016). For these studies, male Wistar rats (230–260 g) were obtained from Envigo Mexico (Mexico City, Mexico). Rats were maintained in individual acrylic cages (27 × 37 × 15 cm) and received chow ad libitum. Enrichment materials included a black acrylic tube, cellulose bedding, and nesting material (GreenSoft. Research Global Solutions, Mexico City, Mexico), and wood chips.

### 4.1. Animal Model

We evaluated the effect of relcovaptan and tolvaptan, antagonists of the V1a and V2 receptors, respectively, in the model of mild thermal dehydration induced in rats (37° C exposure for 1 h on weekdays). We examined the effect of the V1a receptor antagonist relcovaptan (Aobious, Gloucester, MA, USA (intraperitoneal implanted osmotic mini pumps; Alzet Model 2002 (Durect Co, Cupertino CA, USA), constant rate 0.5 L/h, 0.64 mg/day) and the V2 vasopressin receptor antagonist tolvaptan (Medchemexpress LLC, Monmouth Junction, NJ, USA) (subdermal implanted osmotic mini pumps; Alzet Model 2ML2 (Durect Co, Cupertino CA, USA),, constant rate 5 L/h, 0.25 mg/day, [39]).

The specific experimental groups were studied for 2 weeks as follows.

(a) V1a vasopressin receptor blockade (Relcovaptan, 0.64 mg/day)

H-RV group, *n* = 5. Rats were treated with vehicle (dimethyl sulfoxide—DMSO, Sigma-Aldrich St Louis MO, USA), administered in an amount comparable to that of fructose 10% administered to dehydrated rats and tap water the rest of the day; no thermal exposure.

H-R group, *n* = 5. Rats were treated with relcovaptan, administered in an amount comparable to that of fructose (Sigma-Aldrich St Louis MO, USA 10% administered to dehydrated rats and tap water the rest of the day; no thermal exposure.

1. HD-RV group, *n* = 5. Rats were treated with vehicle, followed by two hours of rehydration with fructose 10% after one hour of 37 °C thermal exposure and tap water the rest of the day.
2. HD-R group, *n* = 5. Rats were treated with relcovaptan, followed by two hours of rehydration with fructose 10% after one hour of 37 °C thermal exposure and tap water the rest of the day.

(b) V2 vasopressin receptor blockade (Tolvaptan 0.25 mg/day)

H-TV group *n* = 5. Rat controls were given only vehicle (DMSO + 50% ETOH (Sigma-Aldrich St Louis MO, USA in 0.9% saline) administered in an amount comparable amount to that of fructose 10% administered to dehydrated rats and tap water the rest of the day; no thermal exposure.

H-T group, *n* = 5. Rats were treated with tolvaptan, administered in an amount comparable to that of fructose 10% administered to dehydrated rats and tap water the rest of the day; no thermal exposure.

3. HD-TV group, *n* = 5. Rats were treated with vehicle, followed by two hours of rehydration with fructose 10% after one hour of 37 °C thermal exposure and tap water the rest of the day.
4. HD-T group, *n* = 7. Rats were treated with tolvaptan, followed by two hours of rehydration with fructose 10% after one hour of 37 °C thermal exposure and tap water the rest of the day.

### 4.2. Blood Testing 

Animals were euthanized at the end of the follow-up by deep gas anesthesia and abdominal aortae exsanguination. Both kidneys were washed out with cold PBS, the right kidney was excised, and its cortex and medulla were divided frozen in liquid nitrogen and stored for further processing.

### 4.3. Plasma Testing 

Creatinine and blood urea nitrogen (BUN) were analyzed with enzymatic-based commercial kits (Spin-React, Girona Spain). Copeptin (Peninsula Laboratories, San Carlos, CA, USA), cortisol (Arbor Assays, Ann Arbor MI, USA), and IL-6 (Abcam, Cambridge, UK) were measured by ELISA.

### 4.4. Urine testing

Sixteen-hour urine was collected in metabolic cages starting during the rehydration period at the end of the study. Creatinine was measured with an enzymatic kit (Spin-React, Girona, Spain), and urine protein excretion was measured by Bradford’s method.

### 4.5. Isolation of Tubular Fractions

Tubular fractions were isolated according to published methods [40], and the expression of the marker of tubular damage KIM-1 (GeneTex, Irvine, CA, USA Cat. GTX12016, 1:1000 dilution) was evaluated by western blot. In the non-proximal tubular fraction, cAMP was measured by ELISA (Cayman Chemical, Ann Arbor MI, USA).

### 4.6. Renal Cortex Homogenates

Markers of lipid peroxidation (4-HNE) and protein oxidation were evaluated by methods previously reported [7]. Western blot analyses were performed in three samples randomly chosen from each group for vasopressin receptors V1a (Abcam, Cambridge, UK. ab187753, 1:2000 dilution) and V2 (Abcam, Cambridge, UK, ab109326, 1:1000 dilution), aldose reductase (GeneTex, Irvine, CA, USA Cat. GTX113381, 1:1500 dilution), sorbitol dehydrogenase (GeneTex, Irvine, CA, USA Cat. GTX83588, 1:4000 dilution), fructokinase (GeneTex, Irvine, CA, USA Cat. GTX109591, 1:5000 dilution), renin (GeneTex, Irvine, CA, USA Cat. GTX105734, 1:1500 dilution), angiotensin II (GeneTex, GTX37789, Irvine, CA, USA Cat. 1:1000 dilution), AT-1 receptor (Abcam, Cambridge, UK Cat. ab124734, 1:1500 dilution), and serum/glucocorticoid-regulated kinase 1 (SGK1) (GeneTex, Irvine, CA, USA Cat. GTX54726, 1:3000 dilution); sample normalization based on β-actin (GeneTex, Irvine, CA, USA Cat. GTX109639, 1:10000 dilution) was performed. Proteins of interest and the respective loading controls were run independently at the same time using the same conditions. Bands were visualized using horseradish peroxidase (HRP) secondary antibodies and ECL Clarity (Bio-Rad, Hercules CA, USA). Immunoblots were analyzed using Image Studio Lite 5.2 software (Licor Biosciences, Lincoln, NB, USA).

### 4.7. Statistical Analysis 

To compare the effects of relcovaptan and tolvaptan, the data were normalized as percentage of change using the values of the H-RV and H-TV groups as the 100% values for each drug. Statistical analysis was performed using two-way ANOVA followed by Tukey’s multiple comparisons test. All values presented are expressed as the mean ± SD. Statistical significance was defined as *p* ≤ 0.05. Data was analyzed using GraphPad Prism V 8.3.0 (GraphPad Software, San Diego CA, USA). Raw data for each group are presented in Supplementary Table S1.

## Figures and Tables

**Table 1:** Twenty-four-hour fluid intake, rehydration fluid intake, and body weight loss after heat stress.

Parameter	Relcovaptan				Tolvaptan			
	Hydrated		Heat-dehydrated		Hydrated		Heat-dehydrated	
	H-RV	H-R	HD-RV	HD-R	H-TV	H-T	HD-TV	HD-T
24 h fluid intake (mL/24 h)	32 ± 1	33 ± 1	35 ± 3	34 ± 5	36 ± 2	39 ± 2	35 ± 3	40 ± 5
Rehydration fluid intake (mL/2 h)	8 ± 1	7 ± 2	7 ± 1	7 ± 1	7 ± 2	8 ± 2	8 ± 3	9 ± 1
BW loss after heat stress (% BW *)			2.3 ± 0.1	2.2 ± 1			2.8 ± 1	2.8 ± 1

BW = body weight; H-RV = hydrated relcovaptan vehicle; H-R = hydrated relcovaptan; HD-RV = heat-dehydrated relcovaptan vehicle; HD-R = heat-dehydrated relcovaptan; H-RT = hydrated tolvaptan vehicle; H-T = hydrated tolvaptan; HD-TV = heat-dehydrated tolvaptan vehicle; HD-T = heat-dehydrated tolvaptan. * = Two-way ANOVA: treatment *p* = 0.02; hydration *p* = *ns*; interaction *p* = *ns*. Multiple comparisons were not statistically significant.

**Table 2:** Relcovaptan and tolvaptan partially prevented systemic inflammation and increased cortisol plasma levels induced by heat stress and rehydration with a fructose-containing beverage.

Parameter	Tolvaptan				Relcovaptan			
	Hydrated		Heat-dehydrated		Hydrated		Heat-dehydrated	
	H-RV	H-R	HD-RV	HD-R	H-TV	H-T	HD-TV	HD-T
Plasma Cortisol * pg/mL	25 ± 1	25 ± 2	383 ± 3 ^a,b,c,d^	11 ± 4 ^a,b,c,d,e^	30 ± 5	24 ± 2	298 ± 8 ^a,b,c,d,e,f^	110 ± 4 ^a,b,c,d,e,f,g^
Plasma IL−6 * pg/mL	38 ± 2	37 ± 1	82 ± 3 ^a,b,c,d^	67 ± 2 ^a,b,c,d,e^	38 ± 2	36 ± 2	101 ± 1 ^a,b,c,d,e,f^	75 ± 3 ^a,b,c,d,e,f,g^

H-RV = hydrated relcovaptan vehicle; H-R = hydrated relcovaptan; HD-RV = heat-dehydrated relcovaptan vehicle; HD-R = heat-dehydrated relcovaptan; H-RT = hydrated tolvaptan vehicle; H-T = hydrated tolvaptan; HD-TV = heat-dehydrated tolvaptan vehicle; HD-T = heat-dehydrated tolvaptan. * = Two-way ANOVA: treatment *p* < 0.0001; hydration *p* < 0.0001; interaction *p* < 0.0001. Multiple comparisons: a = *p* < 0.05 vs. H-RV; b = *p* < 0.05 vs. H-R; c = *p* < 0.05 vs. H-TV; d = *p* < 0.05 vs. H-T; e = *p* < 0.05 vs. HD-RV; f = *p* < 0.05 vs. HD-R; g = *p* <0.05 vs. HD-TV.

## Supplementary Materials

Supplementary materials can be found at https://www.mdpi.com/1422-0067/20/22/5764/s1. Table S1: Raw Dataset.
