# Supplementary material for: A Role for Both V1a and V2 Receptors in Renal Heat Stress Injury Amplified by Rehydration with Fructose
Source: Int J Mol Sci. 2019 Nov 16;20(22):5764. doi: 10.3390/ijms20225764 (PMC6888540; doi:10.3390/ijms20225764)
Supplement: Supplementary file 1 [file ijms-20-05764-s001.pdf]

A role for both V1a and V2 receptors in renal heat stress injury amplified by rehydration with fructose.

Supplementary material.

Table S1. Raw dataset

	RELCOVAPTAN				TOLVAPTAN			
	Hydrated		*Heat-dehydrated*		*Hydrated*		*Heat-dehydrated*	
	VEH	RCV	VEH	RCV	VEH	TVP	VEH	TVP
	n=5	n=5	n=5	n=5	n=4	n=5	n=5	n=7
Systolic blood pressure (mmHg)	120 ± 1	118 ± 2	152 ± 1	145 ± 1	118 ± 2	120 ± 2	156 ± 3	139 ± 1
Proteinuria (mg/16 h)	7 ± 0.69	13 ± 2.16	32 ± 1.03	22 ± 1.52	8 ± 0.26	9 ± 1.77	51 ± 2.56	12 ± 0.88
CrCl (ml/min)	1.1 ± 0.10	1.1 ± 0.05	0.6 ± 0.002	0.8 ± 0.03	1.2 ± 0.05	1.1 ± 0.05	0.6 ± 0.02	0.8 ± 0.03
BUN (mg/dL)	16 ± 0.9	17 ± 0.2	39 ± 1.6	28 ± 0.9	19 ± 0.2	20 ± 0.2	31 ± 0.5	26 ± 1.1
Plasma Copeptin (ng/mL)	7 ± 0.9	7 ± 0.2	53 ± 0.3	55 ± 0.4	8 ± 0.1	7± 0.1	48 ± 0.4	46 ± 0.3
**Renal cortex extracts:**								
V1a receptor (AU)	2.6 ± 0.091	2.2 ± 0.009	12.5 ± 0.111	5.6 ± 0.030	0.6 ± 0.001	0.6 ± 0.008	2 ± 0.008	2.0 ± 0.037
V2 receptor (AU)	17.3 ± 0.85	17.2 ± 1.25	24.3 ± 3	26.25 ± 1.30	1.8 ± 0.004	1.5 ± 0.009	4.2 ± 0.005	1.5 ± 0.018
Aldose reductase, AR (AU)	1.42 ± 0.002	1.42 ± 0.002	3.79 ± 0.012	3.37 ± 0.012	2.40 ± 0.004	2.40 ± 0.024	3.25 ± 0.007	2.36 ± 0.003
Sorbitol dehydrogenase, SDH (AU)	4.58 ± 0.079	4.46 ± 0.113	10.15 ± 0.078	10.31 ± 0.042	1.33 ± 0.003	1.34 ± 0.002	3.53 ± 0.072	1.58 ± 0.006
Fructokinase, KHK (AU)	1.08 ± 0.079	1.09 ± 0.002	3.53 ± 0.010	3.54 ± 0.006	4.87 ± 0.01	4.85 ± 0.021	12.56 ± 0.035	5.11 ± 0.019
Renal fructose (mg/mg prot)	6.6 ± 0.3	4.9 ± 0.3	8.7 ± 0.4	6.7 ± 0.3	6.5 ± 0.2	6.1 ± 0.3	8.9 ± 0.3	6.9 ± 0.4
Renal UA (mg/mg prot)	1.4 ± 0.1	1.1 ± 0.1	3.1 ± 0.1	2.6 ± 0.2	0.86 ± 0.03	0.86 ± 0.03	4.3 ± 0.2	2.5 ± 0.2
Oxidized proteins (DNPH, nMOL/mg prot)	3.3 ± 0.3	3.3 ± 0.1	41.9 ± 1.8	28.9 ± 1.8	2.7 ± 0.4	2.5 ± 0.2	24.4 ± 1.5	9.7 ± 0.3
Lipid peroxidation (4HNE, fMol/mg prot)	40 ± 1.2	33 ± 0.4	94 ± 4.5	63 ± 0.9	27 ± 1.2	17 ± 1.1	117 ± 1.2	54 ± 1.9
Renin (AU)	5.17 ± 0.15	5.20 ± 0.15	8.49 ± 0.0.03	6.05 ± 0.02	1.16 ± 0.05	1.12 ± 0.03	2.80 ± 0.03	2.71 ± 0.16
Ang II (AU)	2.82 ± 0.098	2.81 ± 0.045	10.3 ± 0.053	2.30 ± 0.167	0.15 ± 0.010	0.14 ± 0.002	0.45 ± 0.012	0.43 ± 0.021
AT1 receptor (AU)	3.64 ± 0.15	3.43 ± 0.25	10.6 ± 0.22	3.47 ± 0.05	0.53 ± 0.03	0.55 ± 0.01	10.39 ± 0.13	10.21 ± 0.10
Serum/glucocorticoid kinase 1, SGK1 (AU)	2.80 ± 0.07	2.64 ± 0.15	4.58 ± 0.13	2.48 ± 0.06	1.83 ± 0.14	1.72 ± 0.08	6.72 ± 0.11	6.53 ± 0.23
**Tubular extracts:**								
Proximal tubule KIM-1 (AU)	2.34 ± 0.016	2.30 ± 0.046	9.36 ± 0.017	4.70 ± 0.031	3.81 ± 0.008	3.72 ± 0.096	13.23 ± 0.086	2.93 ± 0.012
cAMP non-proximal tubule fraction	0.17 ± 0.02	0.18 ± 0.02	1.17 ± 0.04	2.48 ± 0.02	0.25 ± 0.02	0.17 ± 0.02	0.91 ± 0.02	0.49 ± 0.02

AU= Arbitrary units
